# Survival in dialysis patients is not different between patients with diabetes as primary renal disease and patients with diabetes as a co-morbid condition

**DOI:** 10.1186/1471-2369-12-69

**Published:** 2011-12-19

**Authors:** Marielle A Schroijen, Olaf M Dekkers, Diana C Grootendorst, Marlies Noordzij, Johannes A Romijn, Raymond T Krediet, Elisabeth W Boeschoten, Friedo W Dekker

**Affiliations:** 1 Department of Clinical Epidemiology, Leiden University Medical Center, Albinusdreef 2, 2333 ZA Leiden, the Netherlands; 2 Department of Endocrinology and Metabolic Diseases, Leiden University Medical Center, Albinusdreef 2, 2333 ZA Leiden, the Netherlands; 3 Department of Medical Informatics, Academic Medical Center Amsterdam, Meibergdreef 9, 1105 AZ, Amsterdam, the Netherlands; 4 Department of Nephrology, Academic Medical Center Amsterdam, Meibergdreef 9, 1105 AZ, Amsterdam, the Netherlands; 5 Hans Mak Institute, Koningin Wilhelminalaan 29-B, 1411 EL Naarden, the Netherlands

## Abstract

**Background:**

On dialysis, survival among patients with diabetes mellitus is inferior to survival of non-diabetic patients. We hypothesized that patients with diabetes as primary renal disease have worse survival compared to patients with diabetes as a co-morbid condition and aimed to compare all-cause mortality between these patient groups.

**Methods:**

Data were collected from the Netherlands Cooperative Study on the Adequacy of Dialysis (NECOSAD), a multicenter, prospective cohort study in which new patients with end stage renal disease (ESRD) were monitored until transplantation or death. Patients with diabetes as primary cause of ESRD were compared with patients with diabetes as co-morbid condition and both of these patient groups were compared to patients without diabetes. Analysis was performed using Kaplan-Meier and Cox regression.

**Results:**

Fifteen % of the patients had diabetic nephropathy as primary renal disease (N = 281); 6% had diabetes as co-morbid condition (N = 107) and 79% had no diabetes (N = 1465). During follow-up 42% of patients (N = 787) died. Compared to non-diabetic patients, mortality risk was increased for both patients with diabetes as primary renal disease HR: 1.9 (95% CI 1.6, 2.3) and for patients with diabetes as co-morbid condition HR: 1.7 (95% CI 1.3, 2.2). Mortality was not significantly higher in patients with diabetes as primary renal disease compared to patients with diabetes as co-morbid condition (HR 1.06; 95% CI 0.79, 1.43).

**Conclusions:**

This study in patients with ESRD showed no survival difference between patients with diabetes as primary renal disease and patients with diabetes as a co-morbid condition. Both conditions were associated with increased mortality risk compared to non-diabetic patients.

## Background

Diabetes mellitus is a major contributor to the development of renal failure [1-3]. The proportion of patients with diabetes mellitus that progresses to End Stage Renal Disease (ESRD) is increasing. The increased prevalence of diabetes mellitus is estimated to account for 28% of the increased incidence of renal replacement therapy (RRT) in general [4,5]. A marked difference exists in incidence of patients with ESRD due to diabetic nephropathy between Europe and the United States. The percentage of patients entering RRT because of diabetic nephropathy is 10-15% [5] in Europe compared to 45% in the United States [6].

Survival of diabetic patients and non- diabetic patients with ESRD has improved in the past 10 years [5,7,8]. However, survival among diabetic dialysis patients remains inferior to that of non- diabetic patients [2,9]. Patients with diabetic nephropathy have the largest number of co-morbid conditions within the ESRD population [4]. These conditions are mainly vascular in nature [9-11]. One can hypothesize that in patients with diabetic nephropathy organ damage is not limited to the kidney but also involves other organs resulting in retinopathy, neuropathy and cardiovascular complications. In contrast, patients on dialysis with diabetes as a co-morbid condition may have less pronounced organ damage. Therefore, survival in patients on dialysis with diabetes as co-morbid condition may be better compared to patients with ESRD due to diabetic nephropathy. However, at present this is unknown.

The aim of our present study was therefore to compare survival of dialysis patients with diabetes mellitus as primary cause of the renal failure with dialysis patients with diabetes mellitus as co-morbid condition. Mortality rates in these two groups were compared to mortality rates in dialysis patients without diabetes mellitus. Because of the high incidence of cardiovascular morbidity and mortality in the dialysis population, especially in patients with diabetes, cardiovascular mortality was compared between the three groups. In addition, we performed a stratified analysis according to treatment modality.

## Methods

### Patient selection

Patients who were ≥ 18 years and who began chronic dialysis as the initial renal replacement therapy were eligible for this study. Three months after the start of dialysis was considered as the baseline of present analyses. Informed consent was obtained before inclusion. This study was approved by the Medical Ethics Committees of all participating centres.

### Design

The Netherlands Cooperative Study on the Adequacy of Dialysis (NECOSAD) is a multicenter, prospective cohort study in 38 dialysis centres throughout the Netherlands. New patients with ESRD were included at the time of initiation of dialysis treatment, from January 1, 1997 and were monitored at 3, 6 and thereafter at 6 month intervals until renal transplantation, death or January 1, 2007. Data on demographic characteristics, co-morbidities and primary kidney disease were collected at the time of entry into the study. Dialysis characteristics were collected 3 months after the start of RRT and at 6 month intervals thereafter. At the 3 month visit (baseline) patients were classified according to the treatment modality, i.e. peritoneal dialysis (PD) or hemodialysis (HD). The type and cause of renal disease and causes of death were defined according to the criteria of the European Renal Association- Dialysis and Transplantation Association [12].

### Diabetes mellitus

For the present analysis patients were categorized as follows: 1. patients with diabetic nephropathy as the primary cause of ESRD (diabetes glomerulosclerosis or diabetic nephropathy, type 1 and type 2) [10] and 2. patients with diabetes mellitus as a co-morbid condition, but without diabetic nephropathy as a primary cause of ESRD, and 3. patients with ESRD without diabetes mellitus.

### Study endpoints

The primary endpoint of the present analysis was all cause mortality. Cardiovascular mortality rates were calculated. Cardiovascular mortality was defined as death attributed to myocardial ischemia and infarction, heart failure, cardiac arrest, and cause of death uncertain/not determined [13]. Cause of death uncertain/not determined was considered as cardiovascular death because most of these patients died of a sudden death syndrome and this syndrome had a cardiovascular origin.

### Statistical analysis

Mortality was calculated as incidence rate and expressed as number of deaths/1000 person years. Time to event analysis was performed using Kaplan Meier analysis and the Cox proportional hazard's model. Hazard ratios (HR) were calculated for comparison of all-cause and cardiovascular mortality in the 3 groups. All registered deaths during the follow up period were allocated to treatment modality at the 3 month visit, ignoring modality switches (intention to treat analysis). The multivariate Cox proportional hazards model was extended with adjustments for the possible confounding effects of age and gender. Other clinical characteristics at baseline (such as hypertension, cardiovascular disease) were considered to be potential consequences of diabetes, and thus not used as confounders in multivariate analyses [14]. In an additional analysis the effects of treatment modality (peritoneal dialysis versus hemodialysis) on mortality were studied. All analyses were performed with SPSS statistical software, version 14.0.

## Results

### Patient characteristics

Between January 1997 and January 2007, 1853 patients who survived the first 3 months of dialysis were included. Fifteen percent of patients had diabetes mellitus as primary renal disease, 6% of patients had diabetes as a co-morbid condition whereas the majority of the cohort (79%) had a renal disease without diabetes (Table 1). Patients with diabetes as co-morbid condition were older at baseline (median age 69, range 28-86 y) compared to patients with diabetes as primary renal disease (63, 28-84 y) and patients without diabetes (62, 18-92 y). Retinopathy for which laser coagulation therapy was performed was more frequent in patients with diabetes as primary renal disease compared to patients with diabetes as a co-morbid condition (62% versus 11%). During follow-up 33% of the patients without diabetes received a renal transplant compared to 17% of the patients with diabetes as primary renal disease and 8% of the patients with diabetes as a co-morbid condition.

**Table 1:** Baseline characteristics at 3 months after the start of dialysis.

	*Diabetes as* *primary renal* *disease* *N = 281*	*Diabetes as* *co-morbid* *condition* *N = 107*	*Without* *diabetes* *N = 1465*	P value*
**Age (median yr)**	63 (28-84)	69 (28-86)	62 (18-92)	0.00
**Male gender (%)**	54	58	64	0.01
**Primary renal disease (%)**				
Diabetes Mellitus	100	0	0	
Glomerulonephritis	0	22	24	
Renal Vascular disease	0	22	19	
All other	0	56	58	
**Modality of dialysis (%)**				
HD	64	75	62	0.03
**Comorbidity (%)**				
Myocardial infarction	14	30	11	0.00
Cerebrovascular disease	15	9	7	0.00
Peripheral vascular disease	23	26	12	0.00
Retinopathy (lasercoagulation) (%)	62	11	0	
**Medication: (%)**				
antihypertensive agents	89	79	82	0.01
ACEi, ARBs	36	28	21	0.36
Use of insulin s.c.(%)	75	36	0	
**Blood pressure (mean, mm Hg)**				
Systolic	154 (90-260)	151 (100-210)	148 (90-234)	0.00
Diastolic	79 (44-120)	80 (54-115)	84 (44-145)	0.00
**Smoking (%)**	14	23	24	0.01
**Body Mass Index (kg/m2)**	27 (16-44)	26 (16-45)	25 (15-56)	0.00
**Haemoglobin (g/dl)**	11 (6-16)	11 (6-14)	11 (6-23)	0.11
**Albumin (mmol/l)**	34 (14-47)	35 (13-46)	36 (14-67)	0.02
**Residual GFR (ml/min)**	6	6	5	0.06

*p-value: for continuous variables we used Anova analysis and for binary analysis we used Chi-Square analysis.

### Mortality

**Figure 1:**
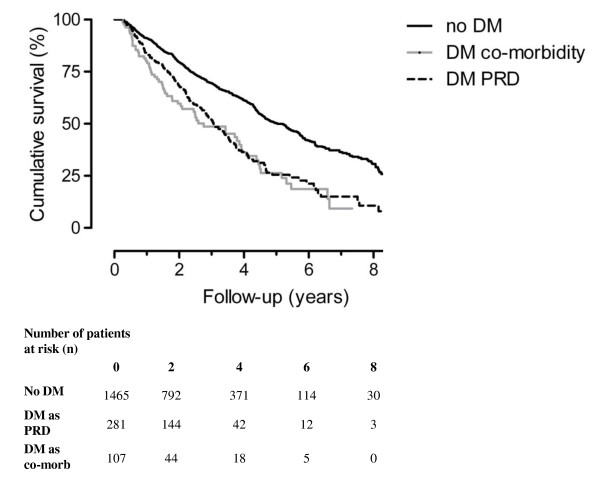
**Kaplan Meier; Survival of patients with diabetes as primary renal disease (DM PRD) compared to patients with diabetes as a co-morbid condition and patients without diabetes mellitus**.

During follow up, 787 patients (42%) of the total group died. The overall mortality rates and cardiovascular mortality rates for each patient group are shown in table 2. Mortality was higher in patients with diabetes as primary renal disease (HR 1.9; 95% CI 1.6, 2.2) and in those with diabetes as a co-morbid condition (2.1, 95% CI 1.6, 2.7) compared to patients without diabetes (Figure 1). After adjustment for age and gender, the HR for patients with diabetes as primary renal disease was 1.9 (95% CI 1.6, 2.3) and 1.7 (95% CI 1.3, 2.2) for patients with diabetes as a co-morbid condition, as compared to non-diabetic patients. Further adjustment for smoking, blood pressure, body mass index, serum albumin, myocardial infarction or stroke, the HR for patients with diabetes as primary renal disease was 1.8 (95% CI 1.3, 2.4) and 1.8 (95% CI 1.5, 2.3) for patients with diabetes as a co-morbid condition, as compared to non-diabetic patients. Also mortality in patients with diabetes as primary renal disease was not clearly higher compared to patients with diabetes as co-morbid condition (HR 1.06; 95% CI 0.79, 1.43).

**Table 2:** Effect of treatment modality on survival; overall mortality and cardiovascular mortality rate on six patient groups.

*Patient group*	*Overall mortality rate* *(Number/* *1000 person years)*	*Cardiovascular mortality rate* *(Number/* *1000 person years) *
No DM	140	41
DM PRD	242	93
DM co-M	288	69

No DM denotes patients without diabetes mellitus

DM PRD denotes patients with diabetes as primary renal disease

DM co-M denotes patients with diabetes as a co-morbid condition

### The effect of treatment modality on survival

**Figure 2:**
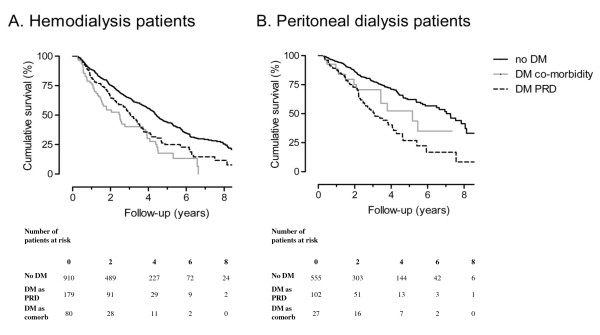
**Kaplan Meier; Survival of patients with diabetes as primary renal disease (DM PRD) versus patients with diabetes as a co-morbid condition and patients without diabetes mellitus in patients on hemodialysis (A) and peritoneal dialysis (B)**.

Thirty-seven percent of patients started on PD (N = 684). Five hundred and fifty five patients had no diabetes, 102 patients had diabetes as primary renal disease and 27 patients had diabetes as a co-morbid condition. After 3 months a few patients switched to hemodialysis; 15 patients without diabetes, 3 patients with diabetes as primary renal disease and none of the patients with diabetes as a co-morbid condition. The highest mortality rate was observed in patients with diabetes as primary renal disease on PD (Figure 2). Following adjustment for age and gender the HR for PD patients with diabetes mellitus as primary renal disease was 2.7 (95% CI 2.0, 3.7) and 1.2 (95% CI 0.7, 2.1) for PD patients with diabetes as a co-morbid condition compared to the reference group of PD patients without diabetes (Table 3).

**Table 3:** Effect of treatment modality on survival; a cox model on six patient groups.

*Patient group*	*Hazard Ratio* *adjusted*	*95% Confidence* *interval*
***Peritoneal dialysis***		
No DM	1.0	2.0, 3.7
DM PRD	2.7	0.7, 2.1
DM co-M	1.2	
***Hemodialysis***		
No DM	1.1	0.9, 1.3
DM PRD	1.8	1.4, 2.3
DM co-M	2.0	1.4, 2.8

Data were adjusted for age and gender

No DM denotes patients without diabetes mellitus

DM PRD denotes patients with diabetes as primary renal disease

DM co-M denotes patients with diabetes as a co-morbid condition

Sixty-three percent (N = 1169) of patients started on HD. Nine hundred and ten patients had no diabetes, 179 patients had diabetes as primary renal disease and 80 patients had diabetes as a co-morbid condition. After 3 months a few patients switched to peritoneal dialysis; 39 patients without diabetes, 3 patients with diabetes as primary renal disease and 5 patients with diabetes as a co-morbid condition. HD patients with diabetes as a co-morbid condition had the highest mortality rates (Figure 2). Adjusted for age and gender the HR for HD patients with diabetes as primary renal disease was 1.8 (95% CI 1.4, 2.3) and 2.0 (95% CI 1.4, 2.8) for HD patients with diabetes as a co-morbid condition compared to the reference group (Table 3). Further adjustment for smoking, blood pressure, body mass index, serum albumin, myocardial infarction or stroke did not materially influence the study results in HD and PD patients. After these adjustments the HR in PD patients with diabetes as primary renal disease was 2.9 (95% CI 2.1, 4.0) and 1.2 (95% CI 0.7, 2.3) for PD patients with diabetes as a co-morbid condition compared to the reference group. The HR in HD patients with diabetes as primary renal disease was 1.7 (95% CI 1.3, 2.3) and 1.9 (95% CI 1.3, 2.7) for HD patients with diabetes as a co-morbid condition compared to the reference group.

## Discussion

In this cohort study we compared survival in patients with ESRD caused by diabetic nephropathy to patients with diabetes as a co-morbid condition and patients without diabetes. Survival in dialysis patients with diabetes was not different between patients with diabetes as primary renal disease and to patients with diabetes as a co-morbid condition. On HD the mortality risk in patients with diabetes as primary renal disease or diabetes as co-morbid condition was increased to a similar extent compared to PD patients without diabetes. Furthermore the mortality risk in PD patients with diabetes as primary renal disease was increased compared to patients without diabetes, whereas this was not the case in PD patients with diabetes as a co-morbid condition.

To our knowledge, this is the first study that investigated mortality in ESRD separately for patients with diabetes as a co-morbid condition and a non-diabetic primary diagnosis of renal disease of different cause. A previous study with a limited number of patients, showed that diabetic patients with a primary diagnosis of adult polycystic kidney disease exhibit a similar survival compared to patients with a primary diagnosis of diabetic nephropathy [15]. Villar et al showed that patients with diabetic nephropathy had a significant worse outcome compared to patients with glomerular nephropathy with a HR of 1.2 [8]. Other studies compared dialysis patients with diabetic nephropathy as primary renal disease to dialysis patients without diabetic nephropathy and showed impaired survival for patients with diabetic nephropathy [6,16]. Present study adds that survival in dialysis patients was not different between patients with diabetes as primary renal disease and patients with diabetes as a co-morbid condition. These results provide important clinical information: diabetes mellitus has a very strong impact on survival even if it is not the primary cause of ESRD.

However, this finding was in contrast with our expectation since we presumed a better prognosis for patients with diabetes as a co-morbid condition compared to patients with diabetes as primary renal disease for the reason that in patients with diabetes as co-morbid condition organ damage due to diabetes mellitus might be less pronounced. In accordance with this notion, at baseline patients with diabetes as a co-morbid condition showed less retinopathy compared to patients with diabetes as a primary renal disease. However the prevalence of myocardial infarction was higher in patients with diabetes as a co-morbid condition, although this was possibly due to different age distribution. A possible explanation for the poor outcome in patients with diabetes as a co-morbid condition could be the additional risk of diabetes in ESRD patients who were already cardiovascular compromised due to their non-diabetic renal disease. Patients with ESRD without diabetes have a high risk of cardiovascular morbidity and mortality [17], just like patients with diabetes mellitus [10,11].

We observed a difference in survival related to treatment modality of ESRD. The mortality risk in PD patients for diabetes as primary renal disease was increased compared to patients without diabetes, whereas this was not the case in PD patients with diabetes as co-morbid condition. The fact that we could not found a difference in PD patients with diabetes as a co-morbid condition could be due to limited power. In PD, dialysis fluids consist of high glucose solutions. These fluids also contained high concentration of glucose degradation products. The peritoneal absorption of glucose degradation products might enhance formation of Advanced Glycosylation End products (AGEs); a non enzymatic reaction of reducing sugars with proteins [18,19]. Accumulation of AGEs is different in PD patients compared to HD patients. A study, determining the influence of dialysis modality on plasma and tissue concentrations of a specific AGE pentosidine, showed that plasma pentosidine levels were significantly lower in PD patients compared with HD patients. In contrast, peritoneal concentrations of pentosidine were significantly higher in patients on PD compared to patients on HD [20]. AGEs may play a role in the pathogenesis of diabetic nephropathy [21]. Therefore accumulation of AGEs might be different in patients with diabetes as primary renal disease as opposed to patients with diabetes as a co-morbid condition. It might be useful to measure serum and peritoneal levels of circulating AGEs in patients with diabetes as primary renal disease compared to patients with diabetes as a co-morbid condition. Probably, PD patients with diabetes as primary renal disease may have had higher levels of (peritoneal) AGEs associated with endothelial dysfunction and atherosclerotic cardiovascular disease [22,23].

There are potential limitations in the present study. First, renal biopsies were not routinely obtained from our patients with a clinical diagnosis of diabetic nephropathy or diabetes as a co-morbid condition. Renal biopsies are the reference standard to confirm whether diabetes is indeed the primary cause of the nephropathy. However a renal biopsy is an invasive procedure with a potential risk of complications and is therefore often not performed in a routine clinical setting. The diagnosis of diabetic nephropathy was a diagnosis by exclusion and was based on the opinion of the physician, reflecting common clinical practice. We can not exclude that some patients could have been misclassified, especially in patients with diabetes as a co-morbid condition and a primary diagnosis of renal vascular disease. In that case it can not be excluded that the diabetes has contributed largely to the renal failure. However exclusion of patients with diabetes as a co-morbid condition and a primary diagnosis of renal vascular disease did not materially influence the study results (data not shown). Second, the number of patients with diabetes either as primary renal disease or as a co-morbid condition was relatively small. Other larger and international studies had to be evaluated to confirm our study results. However, the percentage of patients with diabetes in our cohort was comparable with other studies [5]. Third, glycemic control of our patients was not documented. However treatment of NECOSAD patients was provided according to (inter)national guidelines, and it is unlikely that treatment for diabetes differed between the groups. Fourth, the number of patients who received a renal transplant was higher in patients without diabetes compared to patients with diabetes as primary renal disease or patients with diabetes as a co-morbid condition. Therefore a survival advantage might exist for patients without diabetes mellitus. Finally, some residual confounding by indication might still be present when comparing HD to PD. On peritoneal dialysis survival in patients with diabetes as a co-morbid condition was substantially better compared to patients with diabetes as primary renal disease. Despite the difficulty in categorization of patient groups these data were the best available clinical data. Furthermore, random assignment of treatment modality would hardly be feasible in patients with ESRD. Future prospective analyses are required to determine survival differences in other larger dialysis cohorts between patients with diabetes mellitus as primary renal disease and patients with diabetes as a co-morbid condition, and to establish if hemodialysis or peritoneal dialysis is the optimal treatment regimen for diabetic dialysis patients.

Further we adjusted our analyses for age and gender, while we did not for cardiovascular disease. Cardiovascular disease is most likely on the causal path between diabetes and mortality and should therefore not be adjusted for. Alternatively, it can be speculated that among patients with diabetes as co-morbid condition (if diabetes is not considered as the cause of renal disease), diabetes may also not be the main cause of cardiovascular disease as well. However, exploring this possibility and correcting the main analyses also for cardiovascular disease, did not change the results.

## Conclusions

This study showed that survival in diabetic patients with ESRD was worse compared to non-diabetic patients. Mortality in patients with diabetes as primary renal disease was similar compared to patients with diabetes mellitus as a co-morbid condition. Diabetes mellitus has a very strong impact on survival even if it is not the primary cause of ESRD.

## Competing interests

The authors have had no involvements that might raise the question of bias in the work reported or in the conclusions, implications, or opinions stated. The results presented in this paper have not been published previously in whole or part.

## Authors' contributions

MS performed the statistical analysis and drafted the manuscript. OD, DG, FD have made substantial contributions to acquisition of data, or analysis and interpretation of data. OD, DG, MN, JR, RK, EB, FD, contributed to the conception of the design and revised the manuscript critically. All authors read and approved the final manuscript.

## Pre-publication history

The pre-publication history for this paper can be accessed here:

http://www.biomedcentral.com/1471-2369/12/69/prepub
